# Private schooling and admission to medicine: a case study using matched samples and causal mediation analysis

**DOI:** 10.1186/s12909-015-0415-1

**Published:** 2015-08-20

**Authors:** Muir Houston, Michael Osborne, Russell Rimmer

**Affiliations:** 1 School of Education, University of Glasgow, University Avenue, Glasgow, Scotland UK; 2 Hotel and Tourism Management Institute, Panorama Building, Sörenberg, 6174 Switzerland; 3 School of Arts, Social Sciences and Management, Queen Margaret University, Queen Margaret University Drive, Scotland, UK; 4 School of Education, University of Glasgow, Room 223, St Andrew’s Building, 11 Eldon Street, Glasgow, G3 6NH UK

## Abstract

**Background:**

Are applicants from private schools advantaged in gaining entry to degrees in medicine? This is of international significance and there is continuing research in a range of nations including the USA, the UK, other English-speaking nations and EU countries. Our purpose is to seek causal explanations using a quantitative approach.

**Methods:**

We took as a case study admission to medicine in the UK and drew samples of those who attended private schools and those who did not, with sample members matched on background characteristics. Unlike other studies in the area, causal mediation analysis was applied to resolve private-school influence into direct and indirect effects. In so doing, we sought a benchmark, using data for 2004, against which the effectiveness of policies adopted over the past decade can be assessed.

**Results:**

Private schooling improved admission likelihood. This did not occur indirectly via the effect of school type on academic performance; but arose directly from attending private schools. A sensitivity analysis suggests this finding is unlikely to be eliminated by the influence of an unobserved variable.

**Conclusions:**

Academic excellence is not a certain pathway into medicine at university; yet applying with good grades after attending private school is more certain. The results of our paper differ from those in an earlier observational study and find support in a later study. Consideration of sources of difference from the earlier observational study suggest the causal approach offers substantial benefits and the consequences in the causal study for gender, ethnicity, socio-economic classification and region of residence provide a benchmark for assessing policy in future research.

**Electronic supplementary material:**

The online version of this article (doi:10.1186/s12909-015-0415-1) contains supplementary material, which is available to authorized users.

## Background

Concern about who studies medicine is international. For example, the incidence of private schooling and socioeconomic advantage among those admitted to medicine in Australia, New Zealand and the UK is disproportionate [1]. In South Africa, where race has long been a proxy for disadvantage, black students from private schools have better opportunities than their peers attending state high schools or who are from underprivileged environments [2, 3]. Reflecting this are conclusions for European countries, the USA and Canada that financial barriers can prevent admission to medical degrees, so that studying medicine is an advantage available to those who are already privileged, while precluding the possibility of providing doctors who reflect socio-demographic diversity [4–7].

While researchers report quantitative investigations into private schooling and access to elite degrees, causal analyses were not undertaken [7]. The studies so far have been ‘observational’ in which the selection of individuals into treatment and control groups is not controlled. One issue is that academically more able students may be funnelled into private education, obscuring whether type of school or innate ability, as measured by academic performance, is more important in university admissions decisions. To perform a causal analysis in the case of UK admissions to medical degrees we replicated an experimental design for two groups of applicants that differed only randomly on background characteristics, except that one group attended private schools and the other did not [8, 9]. As far as we can ascertain this has not been done in this much-debated context.

In previous UK research, there is mixed evidence on the association of school type and admission to medicine. In one study, the odds of being accepted onto a medical degree were 89 % greater for applicants from private schools than for comparable applicants from government-funded comprehensives [10]. On the other hand, research by McManus [11, 12] did not identify significant effects of private education on offers to study medicine. However, the point has been made since the 1970s that doctors’ social backgrounds had an impact on the standards of British medical care [13] and decades later, entrants were still dominantly from professional and managerial backgrounds [14]. The research of McManus is part of work covering social class [15], educational qualifications [16, 17], ethnicity [18, 19] and other background factors [20, 21]. He found that some applicants did appear to be disadvantaged, but this was not uniform. Males seemed to be disadvantaged at around half of all medical schools; ethnic minorities were disadvantaged at certain schools, significant socio-economic disadvantage was evident at two medical schools and those applying to nearby medical schools seemed to do better than those who lived outside the area [11].

Our purpose is to undertake the initial causal analysis for 2004, so that the effectiveness of policy interventions in the area of admissions to medicine over the past decade can be assessed in further research. Usually, mediation is assessed by: either estimating an equation and arguing that the effect of a mediator can be estimated by controlling for the effects of other explanators in the equation; or by using an approach associated with Baron and Kenny [9] and extended by subsequent researchers, for example Hayes [22]. However, we wish to control for issues that frequently invalidate conventional approaches such as, omitting relevant variables, unwarranted extrapolation of estimated equations beyond the range of observed data and being dependent on particular modelling assumptions. In such cases, biased estimates can occur and/or invalid conclusions reached on their significance. We employ easy-to-use software that handles these problems [8].

The approach involves two steps. First, using a matching algorithm, samples of applicants from private and public schools are selected on the basis of four background covariates – gender, ethnicity, social class and region of residence when applying for admission to medicine. This reduces the overall sample size but improves ‘balance’ as measured by the extent to which those from private schools have comparable background covariates to a sample who attended non-private schools. Intuitively, if two students have similar background characteristics, but one went to private school and the other did not, the choice to include each in the analysis is made randomly in terms of the covariates available. In this sense, matching moves the analysis towards a random experiment.

**Fig 1:**
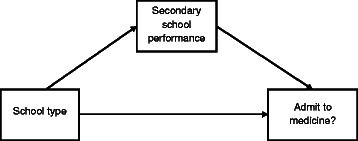
Mediated and direct effects of school type

The second step involves resolution of the overall effect of private schooling into direct and indirect effects on admission, as demonstrated in Fig. 1. The *indirect* or *mediated* effect of interest operates on admissions via secondary school academic performance. The *direct* effect captures other mechanisms linking school type to admission. To obtain the causal effect for an individual, observations are required of receiving treatment (attending private school) and of not doing so (being a control). Obviously, only one state can be observed for each individual, as the unobserved outcome involves being in the opposite state to the one actually experienced. This is overcome by comparing secondary school performances and admissions outcomes for individuals whose pre-treatment characteristics are similar [9]. From these individual effects, unbiased estimates of the average causal mediation effect (ACME) and the average direct effect (ADE) can be simulated for the treatment and control groups provided two *ignorability* assumptions are satisfied. First, for the pre-treatment covariates, assignment to the private-school group should be ignorable in the sense of being statistically independent of potential admissions outcomes and secondary school performance. Second, values of the mediator (secondary school performance) should be ignorable given the observed values of the treatment and pre-treatment covariates.

The first assumption cannot be guaranteed in observational studies and the second assumption cannot be checked even in random experiments [9]. Consequently, a sensitivity analysis is provided to assess how robust our mediation analysis is to violations of ignorability.

The method and data used are set out in the next section. This is followed by sections containing the results of the analysis, a discussion in the context of other research and a conclusion.

## Methods

### Data and measurement

To obtain matched samples of data for causal analysis, anonymised data were drawn from the Universities and Colleges Admissions Service (UCAS). The University of Glasgow Ethics Committee confirmed that studies based on anonymised data have ethical clearance under University regulations.

UCAS data on admissions were coded as one for admission to the study of medicine and zero otherwise. The measure of secondary school performance was taken to be UK tariff score. This provided a quantitative means of comparing academic attainments across the different qualifications in the UK nations and the numbers of subjects taken [10]. Applicants who had completed a degree before applying for admission to medicine were omitted from the analysis. This step was taken as each covariate should precede the attainment of a secondary school performance record [9, 23]. Also omitted were non-UK residents in line with the approach of McManus [11]. Consequently, the unmatched data set was reduced to 6950 applicants aged less than 20. As covariates, we used the applicant characteristics given in the previous section and dichotomous variables were created for eight regions, three ethnicities, three tiers of the UK socioeconomic classification (SEC) and being female.

### Statistical analysis

The package *MatchIt* was used to obtain matched samples of applicants from private and other schools. Like the other software used here, it was written in the *R* programming language, is freely available and well documented [24]. In the following sections we report results of applying propensity score matching and the nearest-neighbour algorithm to select one control match for each treated applicant.

For the matched data, *lm* and *glm* functions from the *R stats* and *MASS* packages were used to estimate equations for log tariff in terms of the covariates and to obtain probit estimation of admission likelihood in terms of the covariates and tariff scores. The *R mediation* software was applied to the regressions to resolve the overall effect of private schooling into direct and indirect effects and to study the sensitivity of results to unobserved confounders (that is, an unobserved covariate that correlates with tariffs and admissions outcomes to such an extent it substantially reduces or eliminates the ACME and ADE).

## Results

### Matching

As shown in the descriptive statistics of Table 1, 1980 individuals or 28.5% of applicants had attended private schools. After matching, all were retained in the treatment group. In the matched controlled sample of size 1980, balance is improved on all covariates except one. For example, the percentage of females in the matched control and treatment groups is closer than is the case for all 4970 control-group members and the treatment group. The exceptional covariate is the Eastern region of England, for which the percentage occurring in the matched control group is the same as for all 4970 controls.

**Table 1:** Descriptive statistics (%) in the original and balanced data

	Treatment group	Control group	
	Private school	Other school	
		All	Matched
*n*	1980	4970	1980
*Percentage of total*	*28.5*	*71.5*	*28.5*
Female	53.2	59.0	52.9
Ethnicity			
Asian	28.8	22.3	29.0
Chinese	4.24	2.39	3.69
White	57.0	64.8	57.6
Other	9.95	10.5	9.65
SEC			
Higher mgr & prof	47.1	34.4	47.5
Lower mgr & prof	25.5	29.1	25.7
Intermediate	10.9	12.7	10.9
Other	16.5	23.8	16.0
Region			
East Midlands	5.86	6.16	6.01
West Midlands	9.29	9.01	9.44
Eastern	7.88	8.03	8.03
Greater London	21.3	17.6	22.1
South East	14.1	12.6	14.1
South West	7.73	7.48	7.63
Wales	3.84	6.04	3.79
Scotland	6.57	4.43	6.31
Other	23.4	28.6	22.6
Log tariff (average value)	0.952	0.912	0.929
Admitted	61.7	51.6	52.9

To obtain the treatment and control groups neither tariff scores nor the admissions outcomes were used [24, 25]. In Table 1, the log of tariff is on average greater among treated applicants than for both the matched control group and all 4970 control cases. Also, being admitted to medicine occurs more frequently for the privately schooled group than for other applicants. The correlations shown in Table 2 for the whole sample of 6950 applicants are all significantly different to zero. However, in the matched sample, the rank correlation between private-school attendance and tariff score is not significantly different to zero.

**Table 2:** Correlations between treatment, mediator and outcome

All 6950 cases	Tariff	Admission
Private school	0.025^b^	0.091^a^
Tariff		0.405^a^
3960 matched cases		
Private school	0.012	0.089^a^
Tariff		0.400^a^

^a, b^denotes significance at better than 1 %, 5 %

### Regressions for tariff and admission

Estimations for tariff scores and admissions outcomes are given in Table 3. In the tariff estimation, private school had little effect. The coefficient was positive, but small and did not exceed the threshold for being significantly different to zero at 5 %, suggesting no evidence of an effect on tariff in 2004. Turning to the admissions columns, tariff score had a positive effect on admission likelihood that is significantly different to zero at better than 1 %. Thus, in samples of applicants matched on gender, ethnicity, SEC and region, private schooling had little effect on tariff, but significantly increased the probability of admission. The reverse of this occurred for applicants of Chinese ethnicity and applicants from Scotland, as they had significantly better tariff scores, but did not have higher probabilities of admission. Another variation is that female applicants had significantly lower tariff scores in 2004, but a higher probability of admission compared with males.

**Table 3:** Estimation of tariff scores and admissions outcomes

	Tariff score		Admissions outcome	
	Coefficient	*t* statistic	Coefficient	*z* statistic
Log(tariff)			1.29	28.88^a^
Private school	0.0197	1.16	0.248	5.64^a^
Female	−0.0345	−2.01^b^	0.114	2.56^b^
White	0.0772	2.55^b^	0.0219	0.28
Asian	−0.00131	−0.04	−0.131	−1.60
Chinese	0.373	7.35^a^	−0.432	−3.27^a^
Higher managerial & professional	0.0598	2.43^b^	0.169	2.66^a^
Intermediate	0.0471	1.40	0.150	1.72
Lower managerial & professional	0.0295	1.09	0.0656	0.94
South East	−0.168	−5.82^a^	0.224	2.98^a^
South West	−0.142	−3.95^a^	0.229	2.46^b^
Greater London	−0.222	−8.47^a^	0.263	3.83^a^
East	−0.0602	−1.72	0.160	1.76
East Midlands	−0.0338	−0.86	0.140	1.37
West Midlands	−0.0287	−0.87	0.109	1.29
Wales	−0.207	−4.45^a^	0.383	3.11^a^
Scotland	0.284	7.44^a^	−0.0620	−0.63
intercept	0.931	23.33^a^	−1.42	−12.52^a^
Residual deviance			4348.1	
Null deviance			5404.5	
Adjusted *R*^2^	0.0762			
*F*	20.33^a^			
Degrees of freedom	16, 3943		17	
*n*	3960		3960	

^a, b^denotes significance at better than 1 %, 5 %

### Causal mediation

In Table 4, average causal mediation effects (ACME) and average direct effects (ADE) are positive. However, the ADEs is greater by around an order of magnitude and the simulated confidence intervals for the ACME contains zero whereas those for the ADE it does not. The positive ADE estimates are consistent with an influence on admission that is not about knowledge or intellectual ability as measured by tariff scores, but presumably is associated with other features of how applicants from private schools navigate the admissions process.

**Table 4:** Causal effects and sensitivity

Average direct and mediation effects (ADE and ACME):	
ADE	0.0794^a^
ACME	0.00805
Overall effect	0.0874^a^
Correlation with an unobserved covariate at which	
ADE = 0	−0.95
ACME = 0	0.40
*n*	3960

^a^95 % confidence interval does not contain zero

Also shown in Table 4 is how large the correlations must be between an unobserved variable and the unexplained parts of tariff scores and admissions outcomes for the causal effects to be zero. For the ACME, the correlation is as low as 0.40. On the other hand, a correlation with magnitude that is more than twice as large is required to invalidate the finding of a positive ADE.

A way to put these results in context is to compare them with other studies using the same approach. The authors do not know of other causal studies in the area of medical-school admissions. However, it is useful to look at how results are interpreted in political psychology by the researchers who wrote the software used for our sensitivity analysis [26]. This context concerns how media presentations translate into political attitudes. The treatment in two studies of this topic was whether subjects read negative news stories; the mediator consisted of levels of anxiety concerning public order; and the outcome consisted of an indicator of political tolerance. Analysis of one research study on media presentations produced a value of 0.34 for the correlation that reduced the effect of the anxiety mediator to zero. An earlier study [27] yielded a correlation of 0.48. The correlation of 0.34 suggests the ACME was somewhat more likely to be eliminated by an unobserved covariate compared with the earlier finding [26].

By contrast, the ADE in our study of medical admissions is positive, the confidence interval does not contain zero and a negative correlation with magnitude 0.95 or above is required for the direct effect of private schooling to be reduced to zero. The question answered in this case is: *Could an unobserved covariate eliminate the direct effect of private schooling on admission to medicine?* The answer is *unlikely*, as the relationship between private schooling and the unobserved covariate would need to be close to perfectly collinear and, because it is negative, would need to detract from the appeal of such candidates to admissions tutors.

On the other hand, the ACME might be reduced to zero by an unobserved covariate, as the required correlation is modest at 0.40, being comparable with the correlations that eliminated ACMEs associated with anxiety. Therefore, it seems that the link from school type to admission via tariffs might be breached. The question asked in this context is *Do more highly achieving secondary school students have unobserved characteristics which influence their likelihood of admission to medical study?* One unobserved characteristic that might have this effect is suitability for dealing with patients, which may not be present generally among high achievers at secondary school. Thus, the answer to the question could be *yes*.

Researchers are advised to undertake mediation analyses in more than one balanced dataset [24]. In this spirit we provide a supplementary file in pdf format (*Additional File**1**.docx*) on another matching algorithm. The same conclusions emerge in this second analysis. It appears that the direct effect detected in the current study is substantial and occurs robustly in matched samples. It is an open question as to why it has not been seen in previous studies. This is taken up in the next section.

## Discussion

In the studies by McManus, Gallagher et al. and us, respectively 30, 26 and 28 % of applications were from private-school students [10, 11]. This is reasonably uniform and a proportion of the variation may be associated with different datasets being extracted from UCAS, which we take up below. Nevertheless the evidence on private-school influence is mixed. Gallagher et al. [10] found for 2006 that the odds of acceptance onto a medical degree were greater for private-school applicants. They employed UCAS data and used the same measure of school achievement as we did. On the other hand, earlier research by McManus for 1996 and 1997 [11, 12] did not identify direct effects of school type on admission. One possible contributor to the difference may be McManus’ use of nine different indicators of school achievement to provide information summarised in later years in tariff scores. What else might explain this difference? McManus had, as dependent variable, receipt of one or more offers to study medicine; Gallagher et al. investigated offers among applicants focused on admission to medicine; we used actual admission or enrolment to study medicine. Broadly, the definitions of McManus and Gallagher et al. are similar; our definition differs in that offers and actual enrolments might diverge for a range of reasons. However, the similarity of our findings to those of Gallagher et al. suggest that in aggregate receiving offers and actually enrolling may not be all that different.

Another difference across the three studies is that the underlying data varied in scope. For McManus, permanent UK residents were studied, including those 21 and over; Gallagher et al. used data for all applicants and ages; and we concentrated on UK residents younger than 21. The different age groups studied might underpin different findings. This is taken up next in the context of comparing the observational studies of McManus and Gallagher et al. with our causal analysis.

McMahon and Gallagher et al. estimate multivariate logistic models. Armed with this type of model, researchers can specify values for the private-school variable and estimate the impact on admissions likelihood, controlling for the covariates. However, when controlling for a large number of covariates, one or both of the treatment and control groups may not contain cases with the exact combination of values to be fixed. This is an aspect of the ‘curse of dimensionality’ [24] and the regression is extrapolated as applying beyond the data range used to estimate it. Such extrapolations beyond observed data are often undetected with the unsuspected effects that regression coefficients are biased and incorrect inferences are drawn [23]. The McManus’ estimations involve upward of 21 variables, with a number converted into collections of categorical indicators [11, 12]; our estimations involve five covariates and individuals with covariate values corresponding to the reference categories do occur in the unmatched and matched data.

Matching on covariates before attempting regression provides a means of avoiding these problems [25]. Estimations are less susceptible to bias, less sensitive to functional forms and to statistical assumptions about the distributions of population values. Further, when matching provides samples of treatment and controls balanced on covariates, estimates of causal effects are relatively unchanged across analyses using different parametric modelling assumptions [25].

Another source of bias in observational studies is that some covariates do not truly precede the treatment. An example is the acquisition of a degree and then applying for admission to medicine, which does not precede the treatment of private schooling or the mediator of obtaining a tariff score. If the intention is to control for a range of covariates, one of which is a post-treatment characteristic, then when type of school changes, the post-treatment indicator may change also, meaning that the effect of the treatment cannot be estimated holding other variables constant [23]. Both McManus and Gallagher et al. allow the possibility of gaining maturity before applying for medicine. To some extent for both, maturity would be attained post-treatment and would confound intentions to estimate effects for one covariate while holding others fixed.

Underlying relationships between covariates might further contribute to the emergence of different findings. In [12], McManus notes that estimating offers to study medicine in terms of school type alone resulted in private school having a positive and significant effect. However, entry of other covariates reversed the sign on private school and the coefficient became non-significant. For other school types, significance and coefficient signs were preserved. This suggests strong inter-correlations between private schooling and other covariates.

A way to think about this is in terms of the potential relationship between private schooling and another covariate such as social class. If in McManus’ data the correlation between these covariates is high, the underlying standard errors for private school may become unusually large and significance is lost. This can arise with little effect on the overall explanatory power of a regression [28]. As an explanation of the different finding on private school, it is tempting to suggest a substantial change in the social classes of applicants by school type for the years covered by McManus, Gallagher et al. and our research. However, such a change, while it may have been in progress in the period from 1996 (the first year of data drawn by McManus) to 2006 (the year data were drawn by Gallagher et al.), probably did not account entirely for a reversal of findings. This would require further investigation as would other sources of inter-correlation and their effects.

A further area of difference in the three studies concerns the treatment of missing values. McManus’ replaced missing values by average values. It is known that this can lead to serious biases in estimated variances and covariances that underpin tests of significance [29]. It would appear Gallagher et al. used listwise deletion although they note missing data is largely associated with overseas and mature applicants and they did not include graduate-entry programmes in their analysis. We both emulated the approach of Gallagher et al. and also used imputation methods designed to avoid the problems associated with use of averages in place of missing values. Our results emerged clearly in both approaches. The imputation methods were not available at the time McManus ran his analysis.

Finally, in the years studied by McManus (1996 and 1997) a direct effect of school type may not have arisen. This would be the case if selection processes functioned differently in those years compared with the studies undertaken in 2004 (by us) and 2006 (by Gallagher et al.). It is possible, but appears unlikely because there has been concern about the backgrounds of doctors since the 1970s [13] and more recently investigators concluded that selection systems can be biased [14, 30, 31]. That is, past research appears to report a history of background and school type influencing admissions.

## Conclusion

Using a causal-mediation approach, the effect of private schooling on admission to medicine operates via two pathways – a direct route and a path via the mediating influence of academic performance. That is, the influence of private schooling is explained only partially by the mediator of academic ability. An area for further research is to explore other features of private schooling that might explain the direct route. There is guidance in the literature [14, 30, 31] on factors that may be relevant such as attention to personal statements and preparation for interviews.

In summing up reasons for different conclusions in one earlier study, it seems the researcher may have been aware of at least some of the concerns given in the previous section [11, 12]. Moreover, remedies we adopted are now readily available, which was not the case when the earlier research was undertaken. Another earlier paper [10] supports our conclusion. The divergence in findings may be associated with different measures of academic performance and a more limited selection of covariates in the later of the two papers. That our results find support in the later paper is likely to rest on our use of a similar performance measure and a limited, but similar collection of covariates. That additionally we establish a direct effect of private schooling on admission to medicine rests on our use of a balanced sample within which unwarranted extrapolation of findings is avoided. Given the evidence of a direct effect in this first causal analysis, the scene is set for investigating whether policies implemented in recent years directly affect selection to study medicine.

## Additional file

Additional file 1: **Additional estimations.** (DOCX 52 kb)
