# Supplementary material for: Private schooling and admission to medicine: a case study using matched samples and causal mediation analysis
Source: BMC Med Educ. 2015 Aug 20;15:136. doi: 10.1186/s12909-015-0415-1 (PMC4545993; doi:10.1186/s12909-015-0415-1)
Supplement: Additional file 1: — Additional estimations. (DOCX 52 kb) [file 12909_2015_415_MOESM1_ESM.docx]

**Additional estimations**

This file contains the results of running an additional matching method on the 2004 UCAS data to that reported in the paper. In addition, as an indicator of robustness, estimations using an ordinal tariff measure are reported also. The statistics describing balance in Tables A1 to A3 were selected based on the preferred approach of [24].

**Table A1 Descriptive and balance statistics for covariates**

|  | **Private school** | | **Other school** | | | | | | | |
| --- | --- | --- | --- | --- | --- | --- | --- | --- | --- | --- |
| **Number of applicants: matching method** | **1980 ^3^** | **Std Dev** | **All (4970)^3^** | **Std dev. Ratio ^4^** | **Genetic ^1^ (4429)^3^:** | **Per cent difference ^5^** | **Std dev. Ratio ^4^** | **Nearest ^2^ (1980)^3^** | **Per cent difference ^5^** | **Std dev. Ratio ^4^** |
| *Female* | 53.2 | 0.499 | 59.0 | 0.99 | 60.5 | -1.5 | 0.98 | 52.9 | 5.5 | 1.00 |
| *Ethnicity* |  |  |  |  |  |  |  |  |  |  |
| Asian | 28.8 | 0.453 | 22.3 | 0.92 | 24.0 | 1.7 | 0.94 | 29.0 | 6.3 | 1.00 |
| Chinese | 4.24 | 0.202 | 2.39 | 0.76 | 1.81 | -0.59 | 0.66 | 3.69 | 1.3 | 0.94 |
| White | 57.0 | 0.495 | 64.8 | 0.97 | 65.1 | -0.30 | 0.96 | 57.6 | 7.2 | 1.00 |
| Other | 9.95 | 0.299 | 10.5 | 1.02 | 9.14 | -0.26 | 0.96 | 9.65 | 0.25 | 0.99 |
| *SEC* |  |  |  |  |  |  |  |  |  |  |
| Higher mgr & prof | 47.1 | 0.499 | 34.4 | 0.95 | 36.8 | 2.4 | 0.97 | 47.5 | 12.3 | 1.00 |
| Lower mgr & prof | 25.5 | 0.436 | 29.1 | 1.04 | 29.4 | -0.26 | 1.05 | 25.7 | 3.4 | 1.00 |
| Intermediate | 10.9 | 0.311 | 12.7 | 1.07 | 12.6 | 0.010 | 1.07 | 10.9 | 1.8 | 1.00 |
| Other | 16.5 | 0.371 | 23.8 | 1.15 | 21.1 | 2.7 | 1.10 | 16.0 | 6.8 | 0.99 |

^1 ‘^Genetic’ refers to genetic matching in which matching based on the propensity score and Mahalanobis distance are optimised [24].

^2^ ‘Nearest’ means nearest-neighbour matching with one match per private-school applicant from among the applicants who attended other schools.

^3^ Percentages in each category

^4^ 100 times the ratio of standard deviations of a characteristic in a sample and for those who attended private school

^5^  The difference of absolute changes in the unmatched data and the difference in the matched data. Each absolute difference is calculated as the difference of the treated percentage minus the relevant unmatched or matched percentage.

**Table A1 (cont.) Descriptive and balance statistics for covariates**

|  | **Private school** | | **Other school** | | | | | | | |
| --- | --- | --- | --- | --- | --- | --- | --- | --- | --- | --- |
| **Number of applicants: matching method** | **1980 ^3^** | **Std Dev** | **All (4970)^3^** | **Std dev. Ratio ^4^** | **Genetic ^1^ (4429)^3^:** | **Per cent difference ^5^** | **Std dev. Ratio ^4^** | **Nearest ^2^ (1980)^3^** | **Per cent difference ^5^** | **Std dev. Ratio ^4^** |
| *Region* |  |  |  |  |  |  |  |  |  |  |
| East Midlands | 5.86 | 0.235 | 6.16 | 1.02 | 6.28 | -0.080 | 1.03 | 6.01 | 0.19 | 1.01 |
| West Midlands | 9.29 | 0.290 | 9.01 | 0.99 | 9.62 | -0.040 | 1.02 | 9.44 | 0.14 | 1.01 |
| Eastern | 7.88 | 0.269 | 8.03 | 1.01 | 8.08 | -0.080 | 1.01 | 8.03 | -0.030 | 1.01 |
| Greater London | 21.3 | 0.410 | 17.6 | 0.93 | 19.4 | 1.8 | 0.97 | 22.1 | 2.9 | 1.01 |
| South East | 14.1 | 0.349 | 12.6 | 0.95 | 13.3 | 0.67 | 0.97 | 14.1 | 1.5 | 1.00 |
| South West | 7.73 | 0.267 | 7.48 | 0.98 | 7.72 | 0.22 | 1.00 | 7.63 | 0.13 | 0.99 |
| Wales | 3.84 | 0.192 | 6.04 | 1.24 | 5.53 | 0.47 | 1.19 | 3.79 | 2.11 | 0.99 |
| Scotland | 6.57 | 0.248 | 4.43 | 0.83 | 4.72 | 0.29 | 0.86 | 6.31 | 1.9 | 0.98 |
| Other | 23.4 | 0.423 | 28.6 | 0.98 | 25.4 | 3.2 | 1.03 | 22.6 | 4.4 | 0.99 |

**Table A2 Balance statistics for the distance measures in the genetic and nearest-neighbour approaches**

|  | Private school | | Other school | | Control | | Per cent balance improvement | | | |
| --- | --- | --- | --- | --- | --- | --- | --- | --- | --- | --- |
|  | Mean | SD | Mean | SD | Mean | SD | Mean difference | eQQ median | eQQ mean | eQQ max |
| Nearest | 0.323 | 0.0839 | 0.270 | 0.108 | 0.323 | 0.0833 | 99.5 | 100.0 | 99.5 | 90.4 |
|  |  |  |  |  |  |  |  |  |  |  |
| Genetic |  |  |  |  | 0.323 | 0.0844 | 99.9 | 25.4 | 36.0 | 30.4 |

**Table A3 Descriptive statistics for tariff scores and admission outcomes**

|  | **Private school** | | **Other school** | | | | | |
| --- | --- | --- | --- | --- | --- | --- | --- | --- |
| **Number of applicants: matching method** | **1980 ^3^** | **Std dev** | **4970 ^3^: unmatched** | **Std dev. ratio ^4^** | **4429 ^3^: genetic ^1^** | **Std dev. ratio ^4^** | **1980 ^3^: nearest ^2^** | **Std dev. ratio ^4^** |
| *Log tariff* | 0.952 ^5^ | 0.542 | 0.912 ^5^ | 1.04 | 0.917 ^5^ | 1.05 | 0.929 ^5^ | 1.04 |
| *Tariff band* 0 to 359 | 18.1 | 0.385 | 21.5 | 1.07 | 21.5 | 1.07 | 21.1 | 1.06 |
| 360 to 419 | 22.2 | 0.416 | 21.4 | 0.99 | 21.0 | 0.98 | 20.3 | 0.97 |
| 420 to 479 | 23.3 | 0.423 | 22.3 | 0.98 | 22.0 | 0.98 | 22.1 | 0.98 |
| 480 to 539 | 19.6 | 0.397 | 18.6 | 0.98 | 18.5 | 0.98 | 19.0 | 0.99 |
| 540 and over | 16.8 | 0.374 | 16.2 | 0.99 | 16.9 | 1.00 | 17.5 | 1.02 |
|  |  |  |  |  |  |  |  |  |
| *Admitted* | 61.7 | 0.486 | 51.6 | 1.03 | 52.0 | 1.03 | 52.7 | 1.03 |

^1 ‘^Genetic’ refers to genetic matching in which matching based on the propensity score and Mahalanobis distance are optimised [24].

^2^ ‘Nearest’ refers to nearest-neighbour matching in which one match per private-school applicant is sought from among the applicants who attended other schools.

^3^ Percentages in each category, unless stated otherwise

^4^ 100 times the standard deviation of a characteristic divided by the standard deviation of the corresponding characteristic for those who attended private school

^5^ Average log value

**Table A4 Estimation of tariff scores**

|  | **Continuous tariff** | | | | **Ordinal tariff** | | | |
| --- | --- | --- | --- | --- | --- | --- | --- | --- |
|  | **Genetic** | | **Nearest** | | **Genetic** | | **Nearest** | |
|  | **Coefficient** | ***t* statistic** | **Coefficient** | ***t* statistic** | **Coefficient** | ***t* statistic** | **Nearest** | ***t* statistic** |
| Private school | 0.012 | 0.82 | 0.020 | 1.16 | 0.002 | 0.07 | 0.023 | 0.67 |
| Female | -0.032 | -2.34* | -0.034 | -2.01* | -0.057 | -2.13* | -0.055 | -1.62 |
| White | 0.102 | 4.29** | 0.077 | 2.55* | 0.173 | 3.70** | 0.134 | 2.23* |
| Asian | -0.019 | -0.77 | -0.001 | -0.04 | -0.049 | -0.99 | -0.013 | -0.02 |
| Chinese | 0.355 | 9.06** | 0.373 | 7.35** | 0.833 | 10.49** | 0.858 | 8.33** |
| Higher managerial & professional | 0.099 | 5.11** | 0.060 | 2.43* | 0.186 | 4.86** | 0.110 | 2.25* |
| Intermediate | 0.074 | 2.78** | 0.047 | 1.40 | 0.143 | 2.73** | 0.089 | 1.33 |
| Lower managerial & professional | 0.046 | 2.12* | 0.030 | 1.09 | 0.078 | 1.84 | 0.040 | 0.74 |
| South East | -0.125 | -5.46** | -0.168 | -5.82** | -0.252 | -5.62** | -0.341 | -5.96** |
| South West | -0.100 | 3.51** | -0.142 | -3.95** | -0.195 | -3.51** | -0.282 | -3.98** |
| Greater London | -0.196 | -9.47** | -0.222 | -8.47** | -0.397 | -9.73** | -0.448 | -8.62** |
| East | -0.050 | -1.80 | -0.060 | -1.72 | -0.096 | -1.75 | -0.123 | -1.77 |
| East Midlands | -0.064 | -2.05* | -0.034 | -0.86 | -0.100 | -1.63 | -0.020 | -0.25 |
| West Midlands | 0.026 | 0.98 | -0.029 | -0.87 | 0.061 | 1.19 | -0.059 | -0.90 |
| Wales | -0.230 | -6.18** | -0.207 | -4.45** | -0.460 | -6.29** | -0.423 | -4.54** |
| Scotland | 0.299 | 9.93** | 0.284 | 7.44** | 0.663 | 10.96** | 0.634 | 8.21** |

^1 ‘^Genetic’ refers to genetic matching in which matching based on the propensity score and Mahalanobis distance are optimised [24].

^2^ ‘Nearest’ refers to nearest-neighbour matching in which one match per private-school applicant is sought from among the applicants who attended other schools.

**, * denotes significance at better than 0.01, 0.05.

**Table A4 (cont.) Estimation of tariff scores**

|  | **Continuous tariff** | | | | **Ordinal tariff** | | | |
| --- | --- | --- | --- | --- | --- | --- | --- | --- |
|  | **Genetic** | | **Nearest** | | **Genetic** | | **Nearest** | |
|  | **Coefficient** | ***t* statistic** | **Coefficient** | ***t* statistic** | **Coefficient** | ***t* statistic** | **Nearest** | ***t* statistic** |
| Cut points |  |  |  |  |  |  |  |  |
| 0\|1 |  |  |  |  | -0.792 | -12.71** | -0.900 | -11.11** |
| 1\|2 |  |  |  |  | -0.170 | -2.75** | -0.249 | -3.10** |
| 2\|3 |  |  |  |  | 0.436 | 7.04** | 0.362 | 4.50** |
| 3\|4 |  |  |  |  | 1.060 | 16.93** | 1.000 | 12.30** |
| intercept | 0.885 | 28.55*** | 0.931 | 23.33*** |  |  |  |  |
| Residual deviance |  |  |  |  | 19999.3 |  | 12355.4 |  |
| Degrees of freedom |  |  |  |  | 6389 |  | 3938 |  |
| Adjusted *R*^2^ | 0.077 |  | 0.076 |  |  |  |  |  |
| *F* | 32.60 |  | 20.33 |  |  |  |  |  |
| Degrees of freedom | 16, 6392 |  | 16, 3943 |  |  |  |  |  |
| *n* | 6409 |  | 3960 |  | 6409 |  | 3960 |  |

**Table A5 Estimation of admissions likelihood**

|  | **Continuous tariff** | | | | **Ordinal tariff** | | | |
| --- | --- | --- | --- | --- | --- | --- | --- | --- |
|  | **Genetic** | | **Nearest** | | **Genetic** | | **Nearest** | |
|  | **Coefficient** | ***z* statistic** | **Coefficient** | ***z* statistic** | **Coefficient** | ***z* statistic** | **Coefficient** | ***z* statistic** |
| Log tariff | 1.27 | 36.39** | 1.29 | 28.88** |  |  |  |  |
| Tariff thresholds |  |  |  |  |  |  |  |  |
| Threshold 2 |  |  |  |  | 1.03 | 18.17** | 1.09 | 15.22** |
| Threshold 3 |  |  |  |  | 1.39 | 24.78** | 1.50 | 20.92** |
| Threshold 4 |  |  |  |  | 1.81 | 29.82** | 1.86 | 24.00** |
| Threshold 5 |  |  |  |  | 2.09 | 32.40** | 2.11 | 25.18** |
| Private school | 0.283 | 7.59** | 0.248 | 5.64** | 0.279 | 7.46** | 0.243 | 5.52** |
| Female | 0.143 | 4.11** | 0.114 | 2.56* | 0.144 | 4.13** | 0.116 | 2.61** |
| White | 0.066 | 1.09 | 0.022 | 0.28 | 0.060 | 0.98 | 0.021 | 0.27 |
| Asian | -0.019 | -0.29 | -0.131 | -1.60 | -0.021 | -0.33 | -0.130 | -1.57 |
| Chinese | -0.342 | -3.39** | -0.432 | -3.27** | -0.336 | -3.29** | -0.404 | -3.04** |
| Higher managerial & professional | 0.172 | 3.46** | 0.169 | 2.66** | 0.174 | 3.49** | 0.172 | 2.70** |
| Intermediate | 0.155 | 2.28* | 0.150 | 1.72 | 0.152 | 2.23* | 0.147 | 1.68 |
| Lower managerial & professional | 0.073 | 1.33 | 0.066 | 0.94 | 0.074 | 1.35 | 0.067 | 0.95 |
| South East | 0.233 | 3.99** | 0.224 | 2.98** | 0.234 | 4.00** | 0.221 | 2.94** |
| South West | 0.247 | 3.40** | 0.229 | 2.46* | 0.243 | 3.34** | 0.223 | 2.39* |
| Greater London | 0.286 | 5.34** | 0.263 | 3.83** | 0.286 | 5.33** | 0.263 | 3.82** |
| East | 0.158 | 2.22* | 0.160 | 1.76 | 0.157 | 2.20* | 0.163 | 1.79 |
| East Midlands | 0.081 | 1.01 | 0.140 | 1.37 | 0.084 | 1.04 | 0.148 | 1.45 |
| West Midlands | 0.065 | 0.98 | 0.109 | 1.29 | 0.062 | 0.93 | 0.105 | 1.24 |

^1 ‘^Genetic’ refers to genetic matching in which matching based on the propensity score and Mahalanobis distance are optimised [24].

^2^ ‘Nearest’ refers to nearest-neighbour matching in which one match per private-school applicant is sought from among the applicants who attended other schools.

**, * denotes significance at better than 0.01, 0.05.

**Table A5 (cont.) Estimation of admissions likelihood**

|  | **Continuous tariff** | | | | **Ordinal tariff** | | | |
| --- | --- | --- | --- | --- | --- | --- | --- | --- |
|  | **Genetic** | | **Nearest** | | **Genetic** | | **Nearest** | |
|  | **Coefficient** | ***z* statistic** | **Coefficient** | ***z* statistic** | **Coefficient** | ***z* statistic** | **Coefficient** | ***z* statistic** |
| Wales | 0.387 | 4.05** | 0.383 | 3.11** | 0.381 | 3.98** | 0.377 | 3.04** |
| Scotland | -0.003 | -0.04 | -0.062 | -0.63 | 0.001 | 0.01 | -0.041 | -0.42 |
| intercept | -1.52 | -17.41** | -1.42 | -12.52** | -1.57 | -17.37** | -1.51 | -12.80** |
| Null deviance | 8817.2 |  | 5404.5 |  | 8817.2 |  | 5404.5 |  |
| Residual deviance | 7118.9 |  | 4348.1 |  | 7106.9 |  | 4338.2 |  |
| Chi square |  |  |  |  |  |  |  |  |
| Degrees of freedom | 17 |  | 17 |  | 20 |  | 20 |  |
| *n* | 6409 |  | 3960 |  | 6409 |  | 3960 |  |

**Table A6 Estimating causal effects**

|  | **Continuous tariff** | | **Ordinal tariff** | |
| --- | --- | --- | --- | --- |
|  | **Genetic** | **Nearest neighbour** | **Genetic** | **Nearest neighbour** |
| **Mediation effects:** |  |  |  |  |
| Control | 0.00493 | 0.00818 | 0.000342 | 0.00481 |
| Treatment | 0.00476 | 0.00792 | 0.000342 | 0.00480 |
| **Direct effects:** |  |  |  |  |
| Control | 0.0898† | 0.0795† | 0.0868† | 0.0752† |
| Treatment | 0.0896† | 0.0792† | 0.0868† | 0.0752† |
| **Overall effects** |  |  |  |  |
| Total | 0.0945† | 0.0874† | 0.0871† | 0.0800† |
| ACME | 0.00485 | 0.00805 | 0.000342 | 0.00480 |
| ADE | 0.0897† | 0.0794† | 0.0868† | 0.0752† |
| **Correlation at which ACME = 0 ^1^** | 0.40 | 0.40 |  |  |
| **Correlation at which ADE = 0 ^1^** | -0.95 | -0.95 |  |  |
| *n* | 6409 | 3960 | 6409 | 3960 |

† 95 per cent confidence interval does not contain zero

^1^ For both treatment and control groups
